# Nitrobenzene as Additive to Improve Reproducibility and Degradation Resistance of Highly Efficient Methylammonium-Free Inverted Perovskite Solar Cells

**DOI:** 10.3390/ma13153289

**Published:** 2020-07-23

**Authors:** Apostolos Ioakeimidis, Stelios A. Choulis

**Affiliations:** Molecular Electronics and Photonics Research Unit, Department of Mechanical Engineering and Materials Science and Engineering, Cyprus University of Technology, 45 Kitiou Kyprianou Street, Limassol 3603, Cyprus; a.ioakeimidis@cut.ac.cy

**Keywords:** perovskite solar cells, methylammonium-free, additives, nitrobenzene, reliability, humidity lifetime, air stability

## Abstract

We show that the addition of 1% (v/v) nitrobenzene within the perovskite formulation can be used as a method to improve the power conversion efficiency and reliability performance of methylammonium-free (CsFA) inverted perovskite solar cells. The addition of nitrobenzene increased power conversion efficiency (PCE) owing to defect passivation and provided smoother films, resulting in hybrid perovskite solar cells (PVSCs) with a narrower PCE distribution. Moreover, the nitrobenzene additive methylammonium-free hybrid PVSCs exhibit a prolonged lifetime compared with additive-free PVSCs owing to enhanced air and moisture degradation resistance.

## 1. Introduction

The power conversion efficiency (PCE) of hybrid perovskite solar cells (PVSCs) has exceeded 25%, reaching that of silicon technology [1]. Nevertheless, the short lifetime and low reproducibility are major obstacles that prevent the commercialization of hybrid perovskite technology. Various degradation protocols are applied to predict the long lifetime of the devices such as continues illumination, heat, humidity and so on [2,3]. Regarding the humidity resistance, the strategies that have been developed to improve the performance include the better encapsulation, application of hydrophobic back contact buffer layers, post treatment of perovskite with functional molecules, use of additive in the perovskite solution, and so on [4,5,6,7,8,9,10]. The use of additives is a simple solution-based approach, and thus a great variety of functional molecules and polymers have been investigated to improve both the efficiency and stability performance of PVSCs [6,11,12,13]. In principle, the various molecules used for the humidity resistance improvement are composed of two main chemical groups, one that can coordinate with the perovskite and the other that can either coordinate with the perovskite or can be hydrophobic [14].

In one of the first reports regarding the use of additive for the enhancement of PVSC, Po-Wei Liang et al. investigated the use of 1,8-diiodooctane (DIO) to improve the film quality by controlling crystallization process (nucleation and growth of crystallites) and by improving perovskite’s morphology and coverage; the PCE of the corresponding devices was increased by ~31% [15]. Xiong Li et al. reported the improvement of PCE and stability of PVSC using phosphonic acid ammonium molecules. It was shown that a strong hydrogen bond was formed between –PO(OH)_2_ of the additive with the perovskite terminal groups –NH_3_^+^, resulting in a smooth and dense perovskite layer. The PCE of the corresponding PVSC incorporating butylphosphonic acid 4-ammonium chloride compare to additive-free PVSCs was doubled and exhibited improved resistance to moisture degradation [16]. In another report, Ignasi Burgués-Ceballos et al. systematically investigated the impact of various additives on the perovskite (commercial precursor ink (I201) from Ossila Ltd.) film morphology and on the performance of PVSCs, concluding that the addition of a small amount (1–5% v/v) of benzaldehyde can increase the PCE by 10% [17]. Since then, several different molecules have been applied for additive engineering of perovskite formulations [18,19,20,21,22,23,24,25,26]. One of the few reports in which methyl ammonium-free perovskite was engineered applying molecular additives is that by Chao Shen et al., who proposed the addition of sulfonyl fluoride-functionalized phenethylammonium in precursor to stabilize FAPbI_3_ perovskite. It was shown that the interaction of sulfonyl group leads to improved crystallinity and passivated surface defects, inducing an increased resistance to moisture invasion. As a result, the additive-based FAPbI_3_ perovskite PVs remained stable in air for more than 1000 h, while the reference devices without additive exhibited a severe reduction in PCE within the first 100 h of the measurement [27].

Another critical issue for controlling the properties of the perovskite formulation is the selection of perovskite composition, which also affects the stability to the various degradation environments [28]. Among them, methylammonium-free (CsFA) perovskite emerges as a potential formulation for efficient and stable PVSC, as it does not incorporate the volatile methylammonium cations [28,29,30,31,32,33,34]. Michael Graetzel et al. has reported that hybrid PVSCs with composition Cs_0.2_FA_0.8_PbI_2.84_Br_0.16_ show a stabilized PCE of unencapsulated devices in air for more than 1000 h [35,36,37].

Here, we report the use of nitrobenzene additive in solution processed hybrid methylammonium-free (CsFA) perovskite as a method to improve PCE, reliability, and air/humidity resistance of inverted PVSCs. The selection of the specific molecule is based on previous reported literature that the nitro group can interact with PbI_6_ cage of the perovskite and can lead to a passivation effects, while the benzene group is a hydrophobic group that has the potential to protect from moisture ingress [38,39]. We show that the addition of nitrobenzene-based additives within the methylammonium-free (CsFA) hybrid perovskite formulation results in increased mean PCE with a much narrower distribution compared with additive-free PVSC under investigation. The increased open circuit voltage (Voc) of hybrid PVSC using 1% (v/v) nitrobenzene additive within the formulation of methylammonium-free (CsFA) indicates the defect passivation. The presented UV–vis measurements on hybrid PVSC precursor solutions that contain nitrobenzene suggest the interaction of perovskite’s colloidal particles with nitrobenzene, while the topography of the nitrobenzene-based PVSC active layer shows a reduced roughness/thickness inhomogeneity as well as passivated grain boundary defects. Moreover, the nitrobenzene additive methylammonium-free (CsFA) hybrid PVSCs retain over 85% of their initial mean PCE after 1500 h in air, whereas the additive-free hybrid PVSCs under investigation decline by more than 65%. Accelerated humidity lifetime testing performed in a humidity chamber at 75% relative humidity (RH) and 22 °C combined with photocurrent mapping measurements has further shown that nitrobenzene-based methylammonium-free (CsFA) inverted PVSCs are more stable owing to the defect passivation and inhibition of moisture permeation effects.

## 2. Materials and Methods

### 2.1. Materials

Pre-patterned glass/Indium Tin Oxide(ITO) substrates (sheet resistance 4 ohm·sq^−1^) were purchased from Psiotec Ltd. (Harpenden, Hertfordshire, UK), PbI_2_ from Alfa Aesar, PC_60_BM from Solenne BV, and PEDOT/PSS (Al 4083) solutions from Heraeus Clevios™ ( Leverkusen, North Rhine-Westphalia, Germany). All the other chemicals used in this study were purchased from Sigma-Aldrich (Munich, Bavaria, Germany).

### 2.2. Hole Transporting Layers’ (HTLs) Preparation on ITO

For the NiOx HTLs, a solution combustion process similar to that of Jae Woong Jung et al. was followed [40]. In details, 1 mmol Ni(NO_3_)_2_·6H_2_O was dissolved in 1 mL 2-methoxy ethanol by stirring for about 15 min. Then, 30.65 μL acetylacetonate was added to the solution and stirred for 1 h in room temperature. ITO substrates were sonicated in acetone and subsequently in isopropanol for 10 min and dried by blow with nitrogen gas before use. The doctor blade technique was applied for the fabrication of the precursor films and the resulting films were dried at 100 °C for 5 min. Subsequently, the obtained films were heated at 300 °C in air for 1 h on a preheated hot plate to complete the combustion process. For PEDOT/PSS (AI 4083), the solution was used without any further treatment. The fabrication of PEDOT/PSS was performed in air, where 50 μL was spin-coated on ITO using the static method at 4000 rpm for 30 s and then annealed at 150 °C for 15 min. After the annealing all films were left to cool down at room temperature for at least 5 min and then transferred into the glovebox.

### 2.3. Devices Fabrication

The inverted PVSC under study was ITO/NiOx/Cs_0.17_FA_.0.83_Pb(I_0.87_Br_0.13_)_3_/PC_60_BM/BCP/Cu. The perovskite solution was prepared in the glovebox by mixing 484.4 mgr PbI_2_, 93.42 mgr PbBr_2_, 186.3 mgr formamidinium iodide (FAI), and 57.6 mgr CsI in 1 mL of 4:1 dimethylformamide (DMF)/dimethyl sulfoxide (DMSO), and steered for 15 min at 70 °C. This solution was split in two equal parts and in one of the two same solutions, 1% v/v of nitrobenzene was added, and then both solutions were steered for 10 more minutes at 70 °C. All the required steps for the PVSC fabrication, after NiOx (PEDOT/PSS) deposition, were performed in the glovebox with >1 ppm O_2_ and >3 ppm H_2_O. The methylammonium free (CsFA) perovskite active layers were fabricated applying deposition parameters, similar to Kelly Schutt et al. [30]. Specifically, the perovskite precursor solution was deposited (45 μL on 1.5 × 1.5 cm substrate) on the NiOx (PEDOT/PSS) and spin-coated for 10 s at 1000 rpm and then for 35 s at 6000 rpm. During the second step and 10 s before the end, 100 μL of chlorobenzene was drop casted on the substrate and the film changed color from bright yellow to brown within the next few seconds. Then, the films were annealed at 80 °C for 1 min on a hot plate followed by 100 °C annealing for 10 min. Next, the substrates were left for 5 min to cool down and PC_60_BM (20 mg/mL in chlorobenzene) solution was dynamically spin coated on the perovskite layer at 1000 rpm for 30 s without any further annealing. The substrates were transferred in a vacuum chamber without being exposed to air and then, under a base pressure of ~5 × 10^−7^ mbar, a thin film of 7 nm bathocuproine (BCP) was deposited. Subsequently, the devices were competed by thermally evaporating 100 nm of copper (Cu) through a shadow mask, resulting in an active area of 0.9 mm^2^. The encapsulation was applied directly after evaporation in the glove box using a glass coverslip and an Ossila E131 encapsulation epoxy resin activated by 365 nm UV irradiation.

### 2.4. Characterizations

For the UV–vis and AFM absorption measurements, the perovskite films were prepared on ITO/NiOx substrates. To perform PL measurements, the perovskite films were prepared (as described in device fabrication section) on quartz substrates, which were treated with UV-O_3_ for 15 min prior to deposition. Absorption measurements on both films and solutions were performed with a Schimadzu UV-2700 UV–vis spectrophotometer. The thickness of the films was measured with a Veeco Dektak 150 profilometer. The current density–voltage (J/V) and Voc-intensity were obtained using a Botest LIV Functionality Test System measured with 10 mV voltage steps and 40 ms of delay time. For illumination, a calibrated Newport Solar simulator equipped with a Xe lamp was used, providing an AM1.5G spectrum at 100 mW cm^−2^ as measured by a certified oriel 91150V calibration cell. A shadow mask was attached to each device prior to measurements to accurately define the corresponding device area. Steady-state photoluminance (PL) experiments were performed on a Fluorolog-3 Horiba Jobin Yvon spectrometer based on an iHR320 monochromator equipped with a visible photomultiplier tube (Horiba TBX-04 module). The PL was non-resonantly excited at 550 nm with the line of a 5 mW Oxxius laser diode. External quantum efficiency (EQE) measurements were performed by Newport System, Model 70356_70316NS. AFM images were obtained using a Nanosurf easy scan 2 controller under the tapping mode. The ageing of the devices was conducted in an environmental chamber.

## 3. Results and Discussion

### 3.1. Perovskite Solar Cells

**Figure 1:**
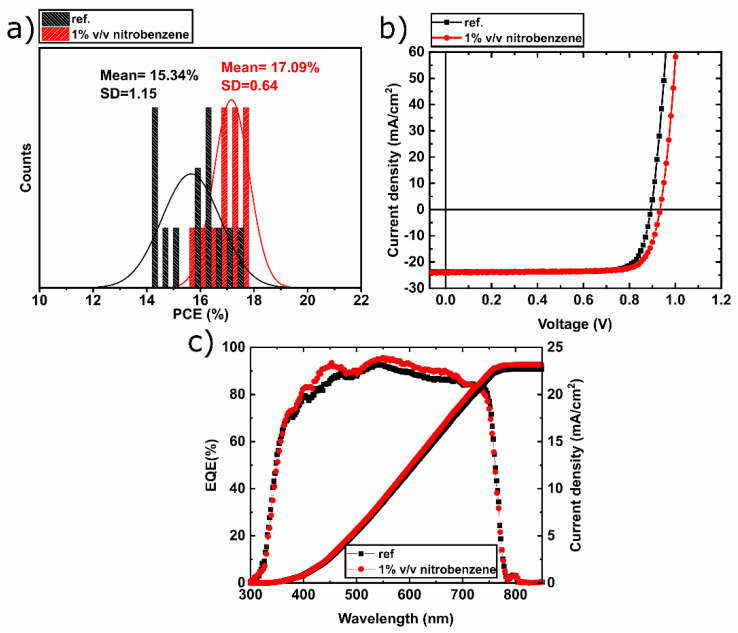
(**a**) The average power conversion efficiency (PCE) and standard deviation (SD) of methylammonium-free (CsFA) hybrid perovskite solar cells (PVSCs) with and without nitrobenzene and the corresponding (**b**) current density–voltage (J–V), (**c**) external quantum efficiency (EQE), and integrated current density of the best performing devices of each batch.

To investigate the additive engineering of methylammonium-free (CsFA) perovskite PVs with nitrobenzene, two batches (12 samples in each batch for over ten repeated runs) of PVSC with the structure glass/ITO/NiOx/Perovskite/PC_60_BM/BCP/Cu were prepared. For the methylammonium free (CsFA) perovskite, active layers were fabricated by applying deposition parameters similar to Kelly Schutt et al., while for the bottom electrode NiOx, hole transporting layers (HTLs) were fabricated on ITO by solution combustion process similar to Jae Woong Jung et al. [30,40]. The PC_60_BM electron transporting layer (ETL) was spin-coated followed by thermal evaporation of bathocuproine (BCP) and copper (Cu) to complete the inverted PVSCs top electrode. Full details are provided within the Materials and Methods section. For the first batch, the perovskite solution was prepared without any additive, while the other batch was prepared with the addition of nitrobenzene in perovskite solution, where for both batches, the used perovskite composition is the methylammonium-free (CsFA) Cs_0.17_FA_.0.83_Pb(I_0.87_Br_0.13_)_3_. By applying different concentrations of nitrobenzene in the methylammonium-free (CsFA) perovskite precursor solution, PCE as a function of additive concentration was investigated and the optimum amount of nitrobenzene additive was identified to be 1% v/v (Figure S1). Figure 1a demonstrates the PCE distributions of the two batches of PVSC with and without 1% nitrobenzene, as well as the extracted mean and standard deviations, respectively. The batch with the nitrobenzene additive exhibits an increased mean PCE value of 17.09% compared with the reference (15.34%) with higher reproducibility, as the respective standard deviation of the former is almost half (0.64%) compared with the last (1.15%). Further, the best performing devices of each batch are presented in Figure 1b and the extracted photovoltaic (PV) parameters are in Table 1. The PVSC with the nitrobenzene delivers Voc = 0.92 V, Jsc = 24.36 mA/cm^2^, and FF = 80.3%, delivering a PCE = 18.02%, while the reference device Voc = 0.89 V, Jsc = 23.99 mA/cm^2^, and FF = 81.3%, delivering a PCE = 17.35%. The integrated current from the external quantum efficiency (EQE) (Figure 1c) is 22.78 and 23.17 mA/cm^2^ for the nitrobenzene and reference device, respectively, consistent with the solar simulator extracted values. The 1% nitrobenzene device shows an enhanced photo response for the wavelengths shorter than 500 nm compared with additive-free reference devices. It will be shown later through optical absorption measurements that nitrobenzene based methylammonium-free (CsFA) perovskite PVs exhibit an increased optical absorption at this range. The experimental results provide evidence that the addition of nitrobenzene results in better control of methylammonium-free (CsFA) perovskite active layer formation, while the observed increase in the reported Voc values indicates passivation of surface defects that consistently resulted in improving device performance reliability.

### 3.2. Perovskite Solutions and Films’ Characterization

**Figure 2:**
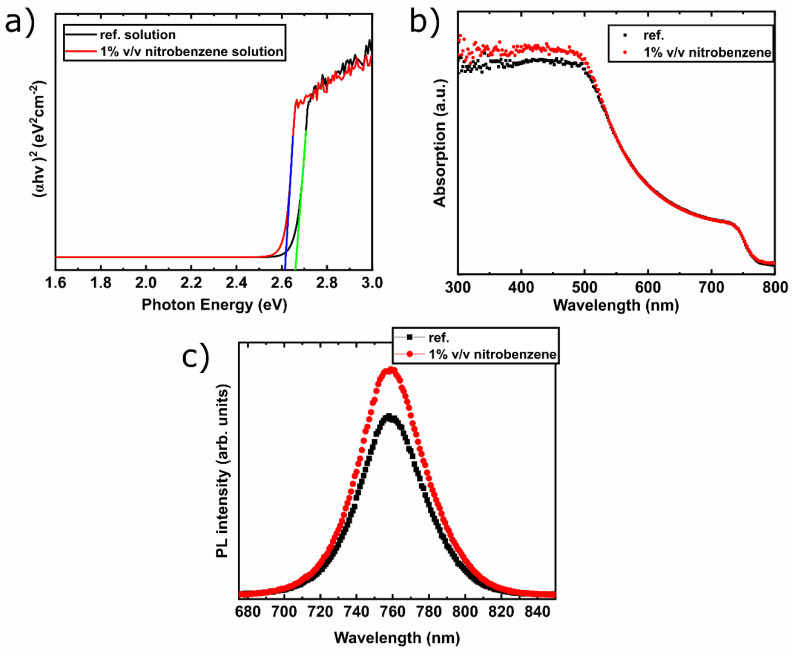
(**a**) Tauc plots of methylammonium-free (CsFA) perovskite with and without 1% v/v nitrobenzene additive calculated from the absorption measurements of precursor solutions and the corresponding (**b**) optical absorption and (**c**) photoluminescence (PL) of the resulting films fabricated on ITO/NiOx/CsFA and glass substrates, respectively.

Further measurements were performed to better clarify the effect of nitrobenzene addition into CsFA PVSCs. To probe the impact of nitrobenzene into the methylammonium-free (CsFA) perovskite formulation, UV–vis absorption measurements were conducted on each precursor solution, with their concentration being two-thirds the concentration used for the PVSC active layer formation in order to let enough light be transmitted through. Figure 2 shows the calculated Tauc-plots of the respective absorption spectra. It is revealed that the absorption band gap of the nitrobenzene-based PVSC formulations are red shifted (2.61 eV) compared with the reference solution (2.66 eV). As has been shown in previous reports, the formed complex of the perovskite precursors with molecules induces a red shift of the absorption edge for the solution under study [41,42,43,44]. Similarly, the observed red shift of the nitrobenzene containing solution compared with the reference is an indication of complex formation between the nitrobenzene additive and the colloidal particles of the perovskite precursor solution. Additional UV–vis measurements (Figure 2b) were performed on the ITO/NiOx/CsFA perovskite structure with and without nitrobenzene, displaying similar spectra at the region up to ~500 nm, while an increase in the absorption of the nitrobenzene containing film was observed for wavelengths shorter than 500 nm. This change is ascribed to reduced light scattering owing to smoother active layer topography rather than to enhanced crystallinity, as the mean size of the grains exhibits a minor change, as shown by the AFM measurements analysis, which is provided below [6,45,46,47,48,49,50]. To study grain boundary passivation effects, photoluminance (PL) (Figure 2c) measurements were performed on methylammonium-free (CsFA) perovskite films fabricated on glass substrates. The PL intensity of nitrobenzene containing methylammonium-free (CsFA) perovskite film exhibits an increased intensity compared with the reference, suggesting less defect mediated charge-carrier recombination (non-radiative process), and thus less grain boundary defects [51].

**Figure 3:**
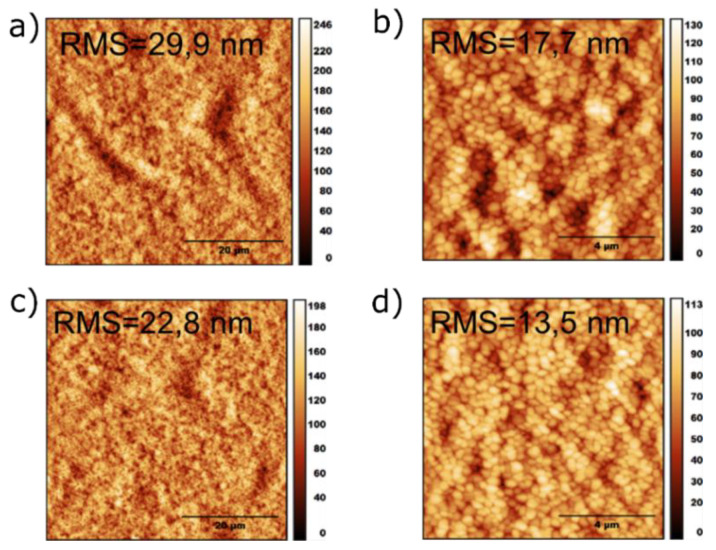
Topography pictures with sizes 50 × 50 μm (**a**,**c**) and 10 × 10 μm (**b**,**d**) obtained with atomic force microscopy (AFM) and the calculated roughness of the (**a**,**b**) reference and (**c**,**d**) nitrobenzene containing methylammonium-free (CsFA) perovskite films fabricated on ITO/NiOx substrates. RMS, root mean square.

To investigate the topography of methylammonium-free (CsFA) perovskite, films were fabricated following the exact same processing conditions that have been applied to corresponding PVSCs on ITO/NiOx substrates with thickness of ~500 nm (determined by profilometry measurements). Atomic force microscopy (AFM) measurements were conducted on the corresponding active layers, with the calculated roughness (root mean square) of the nitrobenzene containing active layer being reduced by ~30% compared with the additive free active layer. Specifically, for the 50 × 50 μm image (Figure 3a,c), the roughness decreases from 29.9 nm to 22.8 nm and, for the 10 × 10 μm image (Figure 3b,d), from 17.7 nm to 13.5 nm. From the grains’ size distribution (Figure S2a,b), it is calculated that the addition of nitrobenzene reduces both the mean grain size and the standard deviation to 304 nm from 329 nm and to 103% from 118%, respectively. Thus, the nitrobenzene containing methylammonium-free (CsFA) perovskite active layers show reduced thickness inhomogeneity and higher compactness, which can be ascribed to retarded crystal growth owing to the formed complex of nitrobenzene with the precursor particles [52,53].

We also examined whether the improved properties of the inverted PVSC were induced by the interaction of the additive with the hole transporting oxide layer (NiOx-underlayer). As NiOx is a nanoparticulate-based functional layer, the high surface area/high number of surface defects could result in high reactivity with nitrobenzene, which might affect the perovskite formation process [54]. Two batches of PVSC were fabricated (with and without nitrobenzene) replacing the NiOx with the PEDOT/PSS as the most common organic hole transporting layer used within inverted PVSC. Consistent with our previously reported experimental evidence using NiOx HTLs, the inverted PVSCs containing nitrobenzene and PEDOT/PSS as HTL also exhibit improved PCE reproducibility with a standard deviation of 0.69% compared with additive-free PVSCs (1.61%), as shown in Figure S3. The above experimental results provide further indication that the origin of the improved performance of the PVSCs is owing to the interaction of the 1% (v/v) nitrobenzene additive with methylammonium-free (CsFA) perovskite precursors rather than nanoparticulate metal-oxide based underlayer effects.

### 3.3. Lifetime Testing of Perovskite Solar Cells

**Figure 4:**
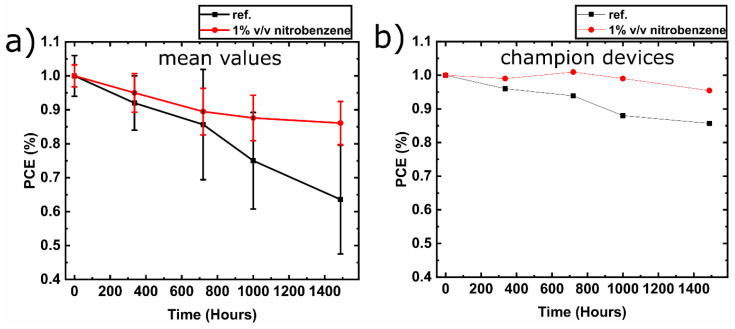
(**a**) Mean PCEs and standard deviations graph of air stability measurements for the encapsulated methylammonium-free (CsFA) PVSC with and without nitrobenzene and (**b**) the corresponding champion devices.

Air stability measurements were performed on methylammonium-free (CsFA) PVSC with and without nitrobenzene, where all devices were encapsulated in inert atmosphere (N_2_) before exposure to air. Figure 4a presents the mean PCE and the standard deviation measurements throughout ageing in ambient conditions. First, it can be observed that the mean PCE of the nitrobenzene containing PVSCs retain around 85% of the initial PCEs after 1500 h, in contrast to the reference PVSCs that decline to approximately 65%. Moreover, the PCE dispersion of reference PVSCs widens significantly during the ageing test compared with nitrobenzene PVSCs. Regarding the champion devices (Figure 4b), the nitrobenzene containing inverted PVSCs exhibit excellent performance, retaining 95% of the initial PCE after 1500 h in air. The results show that the addition of nitrobenzene into methylammonium-free (CsFA) perovskite improves the air stability of the corresponding encapsulated inverted PVSCs.

**Figure 5:**
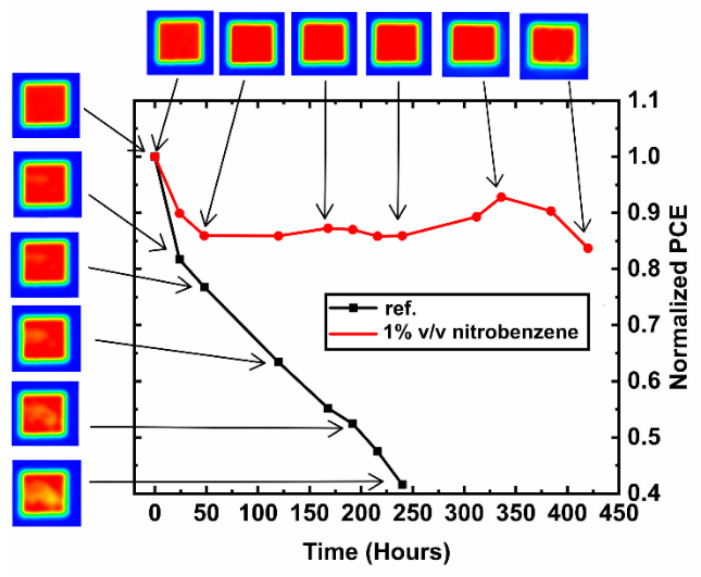
Stability measurements for the encapsulated methylammonium-free (CsFA) perovskite devices with and without nitrobenzene at 75% relative humidity (RH) and 22 °C under dark and the respective photocurrent map of the corresponding devices.

Further to the above air stability measurements, accelerated humidity lifetime testing was also investigated (75 RH% and 22 °C). During this ageing process, device lifetime performance was combined with corresponding photocurrent mapping (PCM) measurements. Figure 5 illustrates the PCE and the corresponding PCM images of the additive-free and nitrobenzene based PVSCs, while the additional normalized PV efficiency parameters Jsc, Voc, and FF as a function of lifetime are presented in Figure S4. The red color of the PCM represents the areas of high photogenerated current, while the yellow to blue color indicates the areas of lower photocurrent. Like the above presented air-stability measurements, the nitrobenzene containing device shows an increased humidity ageing resistance, retaining 85% of its initial PCE for over 400 h. Accordingly, from PCM, it can be seen that the generated photocurrent shows a marginal decrease after 350 h, mostly at the edges of the device area. On the other hand, the additive-free device shows a significant decrease of its initial PCE within the first hours. The yellow spots (reduced photocurrent) at the PCM for the additive-free PVSCs are observed within the first 50 h, followed by a very aggressive photo-current degradation within the next 250 h. The photocurrent mapping observations show good agreement with the recorded normalize Jsc values, which also show an abrupt decrease during accelerated humidity testing at similar time-scales (Figure S4b), whereas the normalized Voc and FF (Figure S4a,c) show a relatively small variance within the presented lifetime performance compared with the initial values. The cause of this degradation can be ascribed to the interaction of perovskite with the H_2_O through the formation of the monohydrate perovskite and then the dihydrate perovskite, which finally leads to its decomposition [55]. However, it remains unclear to us whether the degradation occurs at the interface of perovskite with carrier transporting layers or at the bulk perovskite (or both) [56,57]. It has been reported for other perovskite formulations (e.g., CH_3_NH_3_PbI_3_, FAPbI_3_) that the degradation initiates at the grain boundaries and propagates to the interior [58,59]. Thus, because, in our report, the stoichiometry of perovskite (same perovskite solution is used) and the grain sizes (shown with AFM measurements) are the same for the additive free and nitrobenzene additive based methylammonium-free (CsFA) perovskite, we infer that the origin of the enhanced degradation resistance of the nitrobenzene containing CsFA-based PVSCs is owing to the passivation of perovskite defects, through the reaction of nitrobenzene with the grain boundaries, as well as owing to the inhibition of moisture permeation in the perovskite attributed to the hydrophobic benzene ring [12,57,58,59,60,61,62]. At this initial stage, we have shown that the additive of nitrobenzene can improve the humidity life-time performance of PVSCs under 75% RH and 22 °C conditions. Further ageing tests according to ISOS protocols [3] will be performed in future work.

## 4. Conclusions

In conclusion, the performance of methylammonium-free (CsFA) hybrid PVSCs that incorporate nitrobenzene additive is investigated. We have demonstrated that inverted methylammonium-free (CsFA) PVSCs using 1% v/v nitrobenzene additive provide an increased mean PCE from 15.34% to 17.09%, with a much narrower PCE standard deviation distribution (reduced from 1.15% to 0.64%) compared with corresponding additive-free PVSCs. The improved performance is attributed to the interaction of perovskite’s colloidal particles with nitrobenzene, as well as passivation of grain boundary defects. Importantly, the reported stability of the corresponding encapsulated air-exposed PVSCs under investigation is improved, retaining 85% of the initial PCEs after 1500 h compared with the additive-free devices, which decline to approximately 65% at the same air exposure time scales. Additional accelerated humidity lifetime testing (75% RH and 22 °C) shows that the nitrobenzene 1% v/v containing methylammonium-free (CsFA) inverted hybrid PVSCs exhibit enhanced humidity lifetime performance, retaining 85% of the initial PCE after more than 400 h compared with additive-free PVSCs, which decline within the first 50 h. Although the presented lifetime measurements do not directly correspond to the ISOS-based lifetime-protocols [3], the presented humidity-based accelerated lifetime studies (75% RH and 22 °C) combined with the photocurrent mapping measurements have shown that incorporation of nitrobenzene additive within the formulation of methylammonium-free (CsFA) hybrid inverted PVSCs can be used as a method to improve methylammonium-free (CsFA) hybrid PVSCs’ lifetime performance owing to defect passivation and inhibition of moisture permeation.

## Figures and Tables

**Table 1:** Extracted photovoltaic (PV) parameters from the current density–voltage (J–V) curves of the best performing devices. PCE, power conversion efficiency.

	Voc (V)	Jsc (mA/cm^2^)	FF (%)	PCE (%)
reference	0.89	23.99	81.3	17.35
1% v/v Nitrobenzene	0.92	24.36	80.3	18.02

## Supplementary Materials

The following are available online at https://www.mdpi.com/1996-1944/13/15/3289/s1. See supplementary material for details about Figure S1: PCEs of inverted PVSCs containing different concentrations of nitrobenzene; Figure S2: grain size distribution of active layers with and without nitrobenzene; Figure S3: PCEs of inverted PVSCs using PEDOT/PSS as HTL; Figure S4: normalized PV parameters of PVSCs with and without 1% nitrobenzene during accelerated humidity lifetime testing at 75% RH and 22 °C.

## References

[1] The National Renewable Energy Laboratory (NREL) Best Research-Cell Efficiencies. https://www.nrel.gov/pv/cell-efficiency.html.

[2] Rong Y., Hu Y., Mei A., Tan H., Saidaminov M.I., Seok S.I., McGehee M.D., Sargent E.H., Han H. (2018). Challenges for commercializing perovskite solar cells. Science.

[3] Khenkin M.V., Katz E.A., Abate A., Bardizza G., Berry J.J., Brabec C., Brunetti F., Bulović V., Burlingame Q., Di Carlo A. (2020). Consensus statement for stability assessment and reporting for perovskite photovoltaics based on ISOS procedures. Nat. Energy.

[4] Koushik D., Verhees W.J.H., Kuang Y., Veenstra S., Zhang D., Verheijen M.A., Creatore M., Schropp R.E.I. (2017). High-efficiency humidity-stable planar perovskite solar cells based on atomic layer architecture. Energy Environ.. Sci..

[5] Papadas I.T., Galatopoulos F., Armatas G.S., Tessler N., Choulis S.A. (2019). Nanoparticulate metal oxide top electrode interface modification improves the thermal stability of inverted perovskite photovoltaics. Nanomaterials.

[6] Li B., Fei C., Zheng K., Qu X., Pullerits T., Cao G., Tian J. (2016). Constructing water-resistant CH3NH3PbI3 perovskite films via coordination interaction. J. Mater. Chem. A.

[7] Li X., Xue Z., Luo D., Huang C., Liu L., Qiao X., Liu C., Song Q., Yan C., Li Y. (2018). A stable lead halide perovskite nanocrystals protected by PMMA. Sci. China Mater..

[8] Li N., Zhu Z., Dong Q., Li J., Yang Z., Chueh C.-C., Jen A.K.-Y., Wang L. (2017). Enhanced Moisture Stability of Cesium-Containing Compositional Perovskites by a Feasible Interfacial Engineering. Adv. Mater. Interfaces.

[9] Liu K., Dai S., Meng F., Shi J., Li Y., Wu J., Meng Q., Zhan X. (2017). Fluorinated fused nonacyclic interfacial materials for efficient and stable perovskite solar cells. J. Mater. Chem. A.

[10] Leijtens T., Giovenzana T., Habisreutinger S.N., Tinkham J.S., Noel N.K., Kamino B.A., Sadoughi G., Sellinger A., Snaith H.J. (2016). Hydrophobic Organic Hole Transporters for Improved Moisture Resistance in Metal Halide Perovskite Solar Cells. ACS Appl. Mater. Interfaces.

[11] Hou X., Huang S., Ou-Yang W., Pan L., Sun Z., Chen X. (2017). Constructing Efficient and Stable Perovskite Solar Cells via Interconnecting Perovskite Grains. ACS Appl. Mater. Interfaces.

[12] Yang Z., Dou J., Kou S., Dang J., Ji Y., Yang G., Wu W., Kuang D., Wang M. (2020). Multifunctional Phosphorus-Containing Lewis Acid and Base Passivation Enabling Efficient and Moisture-Stable Perovskite Solar Cells. Adv. Funct. Mater..

[13] Wang R., Xue J., Meng L., Lee J.W., Zhao Z., Sun P., Cai L., Huang T., Wang Z., Wang Z.K. (2019). Caffeine Improves the Performance and Thermal Stability of Perovskite Solar Cells. Joule.

[14] Liu S., Guan Y., Sheng Y., Hu Y., Rong Y., Mei A., Han H. (2019). A Review on Additives for Halide Perovskite Solar Cells. Adv. Energy Mater..

[15] Liang P.W., Liao C.Y., Chueh C.C., Zuo F., Williams S.T., Xin X.K., Lin J., Jen A.K.Y. (2014). Additive enhanced crystallization of solution-processed perovskite for highly efficient planar-heterojunction solar cells. Adv. Mater..

[16] Li X., Ibrahim Dar M., Yi C., Luo J., Tschumi M., Zakeeruddin S.M., Nazeeruddin M.K., Han H., Grätzel M. (2015). Improved performance and stability of perovskite solar cells by crystal crosslinking with alkylphosphonic acid ω -ammonium chlorides. Nat. Chem..

[17] Burgués-Ceballos I., Savva A., Georgiou E., Kapnisis K., Papagiorgis P., Mousikou A., Itskos G., Othonos A., Choulis S.A. (2017). The influence of additives in the stoichiometry of hybrid lead halide perovskites. AIP Adv..

[18] Zuo L., Guo H., DeQuilettes D.W., Jariwala S., De Marco N., Dong S., DeBlock R., Ginger D.S., Dunn B., Wang M. (2017). Polymer-modified halide perovskite films for efficient and stable planar heterojunction solar cells. Sci. Adv..

[19] Chang C.Y., Chu C.Y., Huang Y.C., Huang C.W., Chang S.Y., Chen C.A., Chao C.Y., Su W.F. (2015). Tuning perovskite morphology by polymer additive for high efficiency solar cell. ACS Appl. Mater. Interfaces.

[20] Chiang C.-H., Nazeeruddin M.K., Gratzel M., Wu C.-G. (2017). The synergistic effect of H2O and DMF towards stable and 20% efficiency inverted perovskite solar cells. Energy Environ. Sci..

[21] Rong Y., Hou X., Hu Y., Mei A., Liu L., Wang P., Han H. (2017). Synergy of ammonium chloride and moisture on perovskite crystallization for efficient printable mesoscopic solar cells. Nat. Commun..

[22] Li L., Chen Y., Liu Z., Chen Q., Wang X., Zhou H. (2016). The Additive Coordination Effect on Hybrids Perovskite Crystallization and High-Performance Solar Cell. Adv. Mater..

[23] Wang F., Yu H., Xu H., Zhao N. (2015). HPbI3: A New Precursor Compound for Highly Efficient Solution-Processed Perovskite Solar Cells. Adv. Funct. Mater..

[24] Gong X., Li M., Shi X.-B., Ma H., Wang Z.-K., Liao L.-S. (2015). Controllable Perovskite Crystallization by Water Additive for High-Performance Solar Cells. Adv. Funct. Mater..

[25] De Marco N., Zhou H., Chen Q., Sun P., Liu Z., Meng L., Yao E.-P., Liu Y., Schiffer A., Yang Y. (2016). Guanidinium: A Route to Enhanced Carrier Lifetime and Open-Circuit Voltage in Hybrid Perovskite Solar Cells. Nano Lett..

[26] Deng Y., Zheng X., Bai Y., Wang Q., Zhao J., Huang J. (2018). Surfactant-controlled ink drying enables high-speed deposition of perovskite films for efficient photovoltaic modules. Nat. Energy.

[27] Shen C., Wu Y., Zhang S., Wu T., Tian H., Zhu W.-H., Han L. (2020). Stabilizing Formamidinium Lead Iodide Perovskite by Sulfonyl-Functionalized Phenethylammonium Salt via Crystallization Control and Surface Passivation. Sol. RRL.

[28] Dunfield S.P., Bliss L., Zhang F., Luther J.M., Zhu K., van Hest M.F.A.M., Reese M.O., Berry J.J. (2020). From Defects to Degradation: A Mechanistic Understanding of Degradation in Perovskite Solar Cell Devices and Modules. Adv. Energy Mater..

[29] Turren-Cruz S.-H., Hagfeldt A., Saliba M. (2018). Methylammonium-free, high-performance, and stable perovskite solar cells on a planar architecture. Science.

[30] Schutt K., Nayak P.K., Ramadan A.J., Wenger B., Lin Y.-H., Snaith H.J. (2019). Overcoming Zinc Oxide Interface Instability with a Methylammonium-Free Perovskite for High-Performance Solar Cells. Adv. Funct. Mater..

[31] Chen Y., Yang Z., Jia X., Wu Y., Yuan N., Ding J., Zhang W.-H., Liu S. (2019). (Frank) Thermally stable methylammonium-free inverted perovskite solar cells with Zn2+ doped CuGaO2 as efficient mesoporous hole-transporting layer. Nano Energy.

[32] Zhou Y., Xue H., Jia Y.-H., Brocks G., Tao S., Zhao N. (2019). Enhanced Incorporation of Guanidinium in Formamidinium-Based Perovskites for Efficient and Stable Photovoltaics: The Role of Cs and Br. Adv. Funct. Mater..

[33] Gao X.-X., Luo W., Zhang Y., Hu R., Zhang B., Züttel A., Feng Y., Nazeeruddin M.K. (2020). Stable and High-Efficiency Methylammonium-Free Perovskite Solar Cells. Adv. Mater..

[34] Bush K.A., Frohna K., Prasanna R., Beal R.E., Leijtens T., Swifter S.A., McGehee M.D. (2018). Compositional Engineering for Efficient Wide Band Gap Perovskites with Improved Stability to Photoinduced Phase Segregation. ACS Energy Lett..

[35] Li Z., Yang M., Park J.S., Wei S.H., Berry J.J., Zhu K. (2016). Stabilizing Perovskite Structures by Tuning Tolerance Factor: Formation of Formamidinium and Cesium Lead Iodide Solid-State Alloys. Chem. Mater..

[36] Lee J.W., Kim D.H., Kim H.S., Seo S.W., Cho S.M., Park N.G. (2015). Formamidinium and cesium hybridization for photo- and moisture-stable perovskite solar cell. Adv. Energy Mater..

[37] Yi C., Luo J., Meloni S., Boziki A., Ashari-Astani N., Grätzel C., Zakeeruddin S.M., Röthlisberger U., Grätzel M. (2016). Entropic stabilization of mixed A-cation ABX3 metal halide perovskites for high performance perovskite solar cells. Energy Environ. Sci..

[38] Tremblay M.H., Thouin F., Leisen J., Bacsa J., Srimath Kandada A.R., Hoffman J.M., Kanatzidis M.G., Mohite A.D., Silva C., Barlow S. (2019). (4NPEA)2PbI4 (4NPEA = 4-Nitrophenylethylammonium): Structural, NMR, and Optical Properties of a 3 × 3 Corrugated 2D Hybrid Perovskite. J. Am. Chem. Soc..

[39] Cheng Y.J., Yang S.H., Hsu C.S. (2009). Synthesis of conjugated polymers for organic solar cell applications. Chem. Rev..

[40] Jung J.W., Chueh C.C., Jen A.K.Y. (2015). A Low-Temperature, Solution-Processable, Cu-Doped Nickel Oxide Hole-Transporting Layer via the Combustion Method for High-Performance Thin-Film Perovskite Solar Cells. Adv. Mater..

[41] McMeekin D.P., Wang Z., Rehman W., Pulvirenti F., Patel J.B., Noel N.K., Johnston M.B., Marder S.R., Herz L.M., Snaith H.J. (2017). Crystallization Kinetics and Morphology Control of Formamidinium–Cesium Mixed-Cation Lead Mixed-Halide Perovskite via Tunability of the Colloidal Precursor Solution. Adv. Mater..

[42] Rahimnejad S., Kovalenko A., Forés S.M., Aranda C., Guerrero A. (2016). Coordination Chemistry Dictates the Structural Defects in Lead Halide Perovskites. Chem. Phys. Chem.

[43] Guo X., McCleese C., Kolodziej C., Samia A.C.S., Zhao Y., Burda C. (2016). Identification and characterization of the intermediate phase in hybrid organic-inorganic MAPbI3 perovskite. Dalt. Trans..

[44] Yan K., Long M., Zhang T., Wei Z., Chen H., Yang S., Xu J. (2015). Hybrid Halide Perovskite Solar Cell Precursors: Colloidal Chemistry and Coordination Engineering behind Device Processing for High Efficiency. J. Am. Chem. Soc..

[45] Wu T., Wu J., Tu Y., He X., Lan Z., Huang M., Lin J. (2017). Solvent engineering for high-quality perovskite solar cell with an efficiency approaching 20%. J. Power Sources.

[46] Fei C., Guo L., Li B., Zhang R., Fu H., Tian J., Cao G. (2016). Controlled growth of textured perovskite films towards high performance solar cells. Nano Energy.

[47] Cao X., Zhi L., Li Y., Fang F., Cui X., Ci L., Ding K., Wei J. (2018). Fabrication of Perovskite Films with Large Columnar Grains via Solvent-Mediated Ostwald Ripening for Efficient Inverted Perovskite Solar Cells. ACS Appl. Energy Mater..

[48] Fei C., Li B., Zhang R., Fu H., Tian J., Cao G. (2017). Highly Efficient and Stable Perovskite Solar Cells Based on Monolithically Grained CH3NH3PbI3 Film. Adv. Energy Mater..

[49] Liu C., Huang Z., Hu X., Meng X., Huang L., Xiong J., Tan L., Chen Y. (2018). Grain Boundary Modification via F4TCNQ to Reduce Defects of Perovskite Solar Cells with Excellent Device Performance. ACS Appl. Mater. Interfaces.

[50] Chaudhary B., Koh T.M., Febriansyah B., Bruno A., Mathews N., Mhaisalkar S.G., Soci C. (2020). Mixed-Dimensional Naphthylmethylammoinium-Methylammonium Lead Iodide Perovskites with Improved Thermal Stability. Sci. Rep..

[51] Zhang H., Ren X., Chen X., Mao J., Cheng J., Zhao Y., Liu Y., Milic J., Yin W.J., Grätzel M. (2018). Improving the stability and performance of perovskite solar cells: Via off-the-shelf post-device ligand treatment. Energy Environ. Sci..

[52] Wu Y., Islam A., Yang X., Qin C., Liu J., Zhang K., Peng W., Han L. (2014). Retarding the crystallization of PbI 2 for highly reproducible planar-structured perovskite solar cells via sequential deposition. Energy Environ. Sci..

[53] Lee J.W., Bae S.H., Hsieh Y.T., De Marco N., Wang M., Sun P., Yang Y. (2017). A Bifunctional Lewis Base Additive for Microscopic Homogeneity in Perovskite Solar Cells. Chem.

[54] Mei A., Li X., Liu L., Ku Z., Liu T., Rong Y., Xu M., Hu M., Chen J., Yang Y. (2014). A hole-conductor-free, fully printable mesoscopic perovskite solar cell with high stability. Science.

[55] Howard J.M., Tennyson E.M., Barik S., Szostak R., Waks E., Toney M.F., Nogueira A.F., Neves B.R.A., Leite M.S. (2018). Humidity-Induced Photoluminescence Hysteresis in Variable Cs/Br Ratio Hybrid Perovskites. J. Phys. Chem. Lett..

[56] Barbé J., Newman M., Lilliu S., Kumar V., Lee H.K.H., Charbonneau C., Rodenburg C., Lidzey D., Tsoi W.C. (2018). Localized effect of PbI2 excess in perovskite solar cells probed by high-resolution chemical-optoelectronic mapping. J. Mater. Chem. A.

[57] Wang Q., Chen B., Liu Y., Deng Y., Bai Y., Dong Q., Huang J. (2017). Scaling behavior of moisture-induced grain degradation in polycrystalline hybrid perovskite thin films. Energy Environ. Sci..

[58] Yun J.S., Kim J., Young T., Patterson R.J., Kim D., Seidel J., Lim S., Green M.A., Huang S., Ho-Baillie A. (2018). Humidity-Induced Degradation via Grain Boundaries of HC(NH2)2PbI3 Planar Perovskite Solar Cells. Adv. Funct. Mater..

[59] Wang M., Fu Q., Yan L., Guo P., Zhou L., Wang G., Zheng Z., Luo W. (2019). Improving the Performance and Reproducibility of Inverted Planar Perovskite Solar Cells Using Tetraethyl Orthosilicate as the Antisolvent. ACS Appl. Mater. Interfaces.

[60] Yun S.C., Ma S., Kwon H.C., Kim K., Jang G., Yang H., Moon J. (2019). Amino acid salt-driven planar hybrid perovskite solar cells with enhanced humidity stability. Nano Energy.

[61] Wu T., Liu X., He X., Wang Y., Meng X., Noda T., Yang X., Han L. (2020). Efficient and stable tin-based perovskite solar cells by introducing π-conjugated Lewis base. Sci. China Chem..

[62] Wang F., Geng W., Zhou Y., Fang H.H., Tong C.J., Loi M.A., Liu L.M., Zhao N. (2016). Phenylalkylamine Passivation of Organolead Halide Perovskites Enabling High-Efficiency and Air-Stable Photovoltaic Cells. Adv. Mater..

