# Supplementary material for: Nitrobenzene as Additive to Improve Reproducibility and Degradation Resistance of Highly Efficient Methylammonium-Free Inverted Perovskite Solar Cells
Source: Materials (Basel). 2020 Jul 23;13(15):3289. doi: 10.3390/ma13153289 (PMC7435988; doi:10.3390/ma13153289)
Supplement: Supplementary file 1 [file materials-13-03289-s001.pdf]

Supplementary Information

Nitrobenzene as additive to improve reproducibility and degradation resistance of highly efficient methylammonium-free inverted perovskite solar cells

Apostolos Ioakeimidis[1] and Stelios. A. Choulis[1]*

[1]Molecular Electronics and Photonics Research Unit, Department of Mechanical Engineering and Materials Science and Engineering, Cyprus University of Technology, 45 Kitiou Kyprianou Street, Limassol, 3603, Cyprus

* Corespondence: stelios.choulis@cut.ac.cy

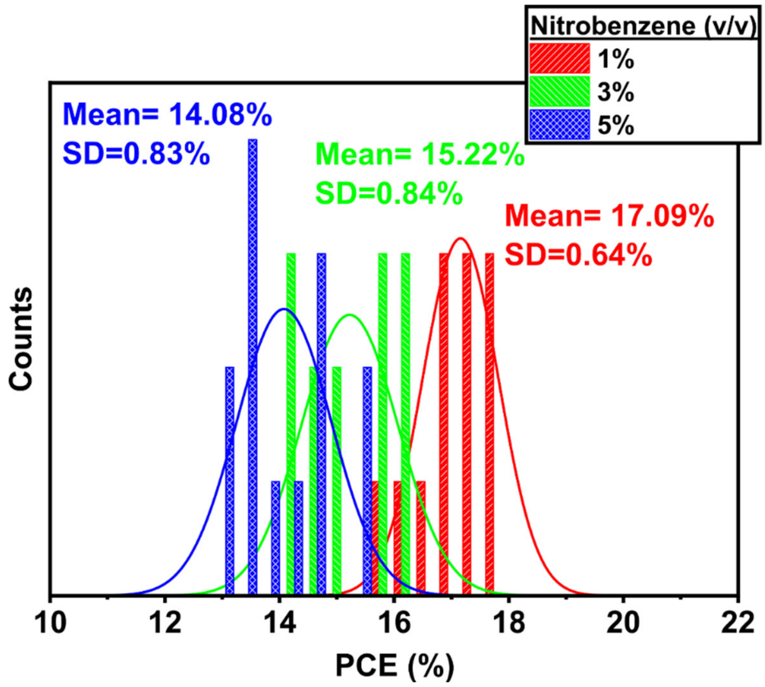

Figure S1 mean PCE and standard deviation (SD) of PVSCs with different concentrations of nitrobenzene.

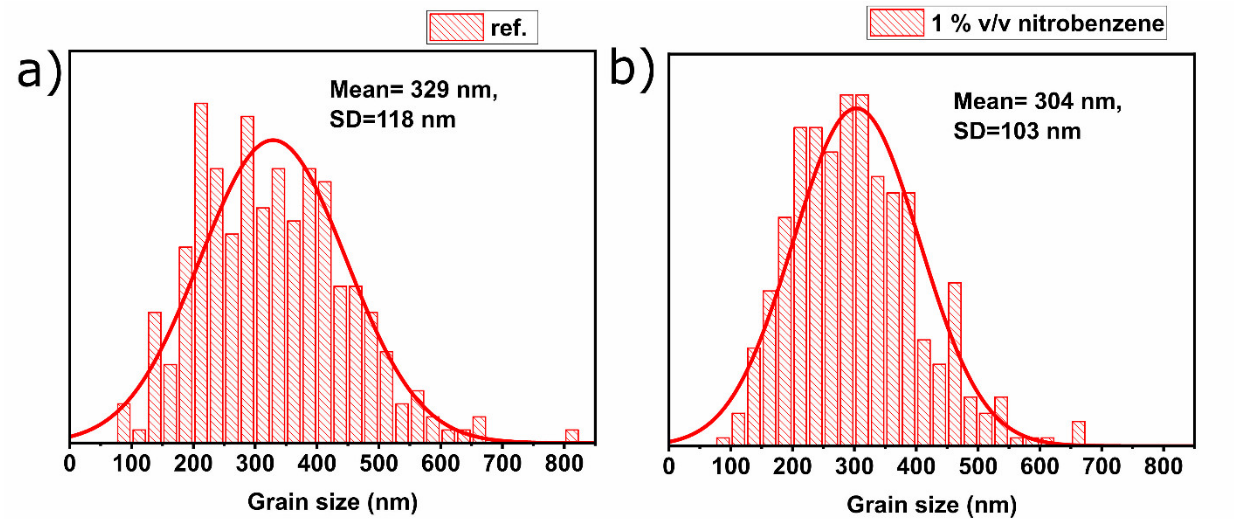

Figure S2 Grain size distribution of pristine and nitrobenzene containing perovskite films fabricated on top of ITO/NiOx substrates

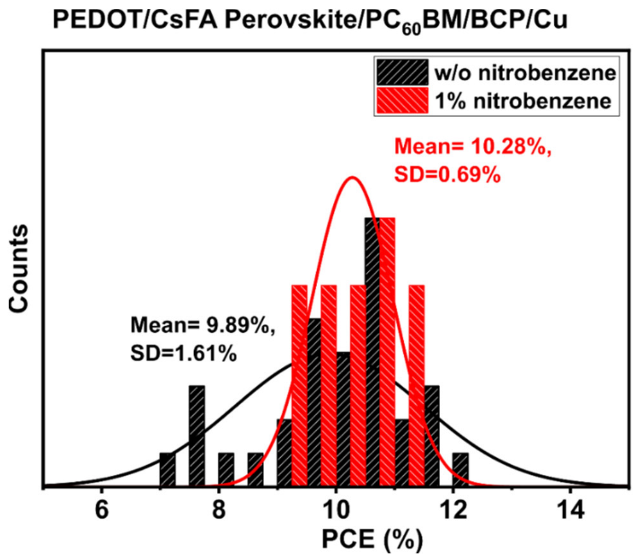

Figure S3 mean PCE and standard deviation (SD) of PVSC with and without nitrobenzene fabricated on ITO/PEDOT:PSS substrate.

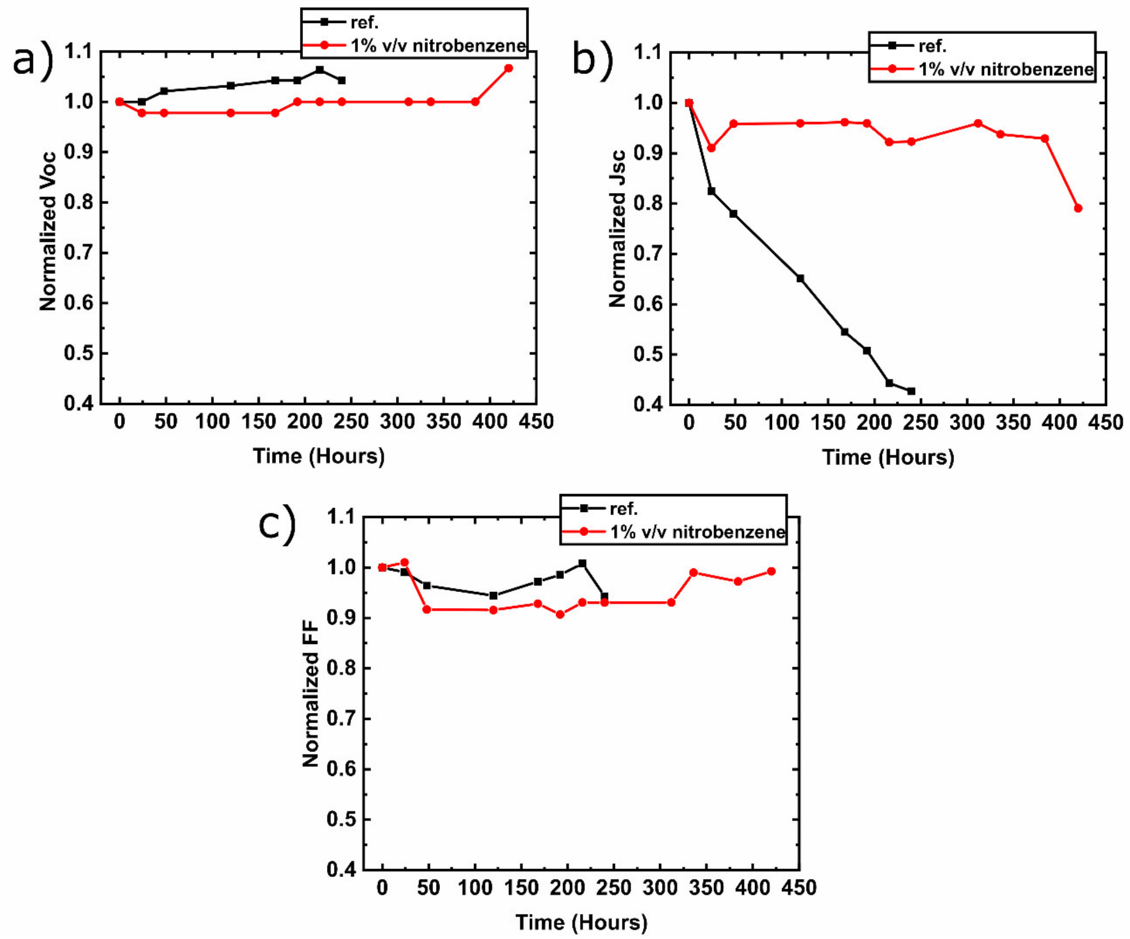

Figure S4 The normalized PV parameters current density (Jsc), Open circuit voltage (Voc) and fill factor (FF) of the corresponding PVSC with and without 1% nitrobenzene during accelerated lifetime testing
